# Impact of surgical urgency on outcomes after minimally invasive coronary artery bypass surgery: a retrospective cohort study

**DOI:** 10.1186/s13019-025-03768-1

**Published:** 2025-12-24

**Authors:** Hemn Abdulrahman Abdullah, Darya Nadir Saeed, Abdullah Hayder Flaih, Marwan Abdullah Barzani

**Affiliations:** 1 Department of Cardiovascular and Thoracic Surgery, Ministry of Health, Erbil, Iraq; 2 Department of Biology, College of sciences, Salahaddin University-Erbil, Erbil, Iraq; 3 Hawler Teaching Hospital, Ministry of Health, Erbil, Iraq

**Keywords:** Surgical urgency, Coronary artery bypass grafting, Minimally invasive cardiac surgery, Postoperative outcomes, Vasopressor support, Renal dysfunction, Acute respiratory failure.

## Abstract

**Background:**

Surgical urgency in minimally invasive coronary artery bypass grafting (MICS-CABG) is associated with increased perioperative risk. This study evaluates the impact of surgical urgency on early postoperative outcomes in patients undergoing isolated MICS-CABG.

**Methods:**

A retrospective cohort study was conducted at Shar Hospital, Erbil, Iraq, including 311 patients who underwent isolated MICS-CABG between September 2021 and December 2024. Patients were classified as elective group (*n* = 285) and urgent group (*n* = 26) according to Society of Thoracic Surgeons criteria. Baseline demographics, intraoperative variables, and early postoperative outcomes were compared using appropriate statistical tests with a significance level of *p* < 0.05.

**Results:**

Baseline characteristics, including, gender, and body mass index, showed no significant differences between elective and urgent groups. However, the urgent group had significantly lower preoperative left ventricular ejection fraction (49.2% ± 12.0 vs. 53.8% ± 10.0, *p* = 0.032) and higher preoperative serum creatinine levels (1.59 ± 1.58 vs. 1.19 ± 1.94 mg/dL, *p* = 0.008). Intraoperatively, urgent patients required more vasopressor support (adrenaline: 26.9% vs. 10.2%, *p* = 0.020; noradrenaline: 73.1% vs. 40.7%, *p* = 0.001). Postoperatively, urgent cases had higher serum creatinine (1.86 ± 1.79 vs. 1.14 ± 0.58 mg/dL, *p* = 0.037) and more frequent incidence of acute respiratory failure in urgent group (39.1% vs. 17.5%, *p* = 0.023).

**Conclusions:**

Urgent MICS-CABG was associated with increased vasopressor use, renal dysfunction, and respiratory complications.

**Trial registration:**

Not applicable.

## Background

Surgical urgency is a well-established determinant of patient outcomes, with urgent procedures consistently associated with increased perioperative risks compared to elective surgeries [1, 2]. These risks are often attributed to limited time for preoperative optimization and the severity of underlying clinical conditions [3].

In the context of cardiothoracic surgery, urgency plays a particularly critical role. Coronary artery bypass grafting (CABG) performed under urgent conditions—most commonly in response to acute coronary syndrome —has been linked to higher rates of postoperative complications, including morbidity and mortality, especially when compounded by hemodynamic instability or other comorbidities [4, 5]. Data from the SWEDEHEART registry and other large-scale studies underscore the importance of comprehensive preoperative assessment in urgent cases to mitigate these risks and improve surgical outcomes [6].

Although urgent CABG is inherently associated with elevated risk profiles, several studies have demonstrated that with timely intervention and optimized perioperative care, patients may still achieve favorable outcomes and substantial improvements in quality of life [7–10]. These findings support the continued use of urgent CABG in high-risk settings, while also emphasizing the need for careful patient selection and surgical planning.

With the growing adoption of minimally invasive coronary artery bypass grafting (MICS-CABG) as an alternative to conventional open-chest surgery, questions remain about how surgical urgency influences outcomes in this subset of procedures. MICS-CABG offers benefits such as reduced surgical trauma, shorter hospital stays, and faster recovery; however, evidence is limited regarding the safety and effectiveness of this technique in urgent settings.

Therefore, this study aims to evaluate the impact of surgical urgency on outcomes following MICS-CABG, specifically by comparing postoperative complications and overall clinical outcomes between urgent and elective procedures. By addressing this gap, the study seeks to provide data-driven insights that can guide clinical decision-making and optimize timing strategies for patients requiring coronary revascularization.

## Methods

### Study design and setting

A retrospective cohort study was conducted at the cardiovascular surgery unit of Shar Hospital, Erbil, Iraq. The study included patients who underwent MICS-CABG between September 2021 and December 2024. Patients were retrospectively stratified into two groups—elective and urgent—based on the urgency classification defined by the Society of Thoracic Surgeons (STS), where elective cases were those scheduled in a stable clinical state, and urgent cases required surgery during the same hospitalization due to clinical deterioration without meeting emergent criteria within 48 h.

## Study population

**Fig. 1:**
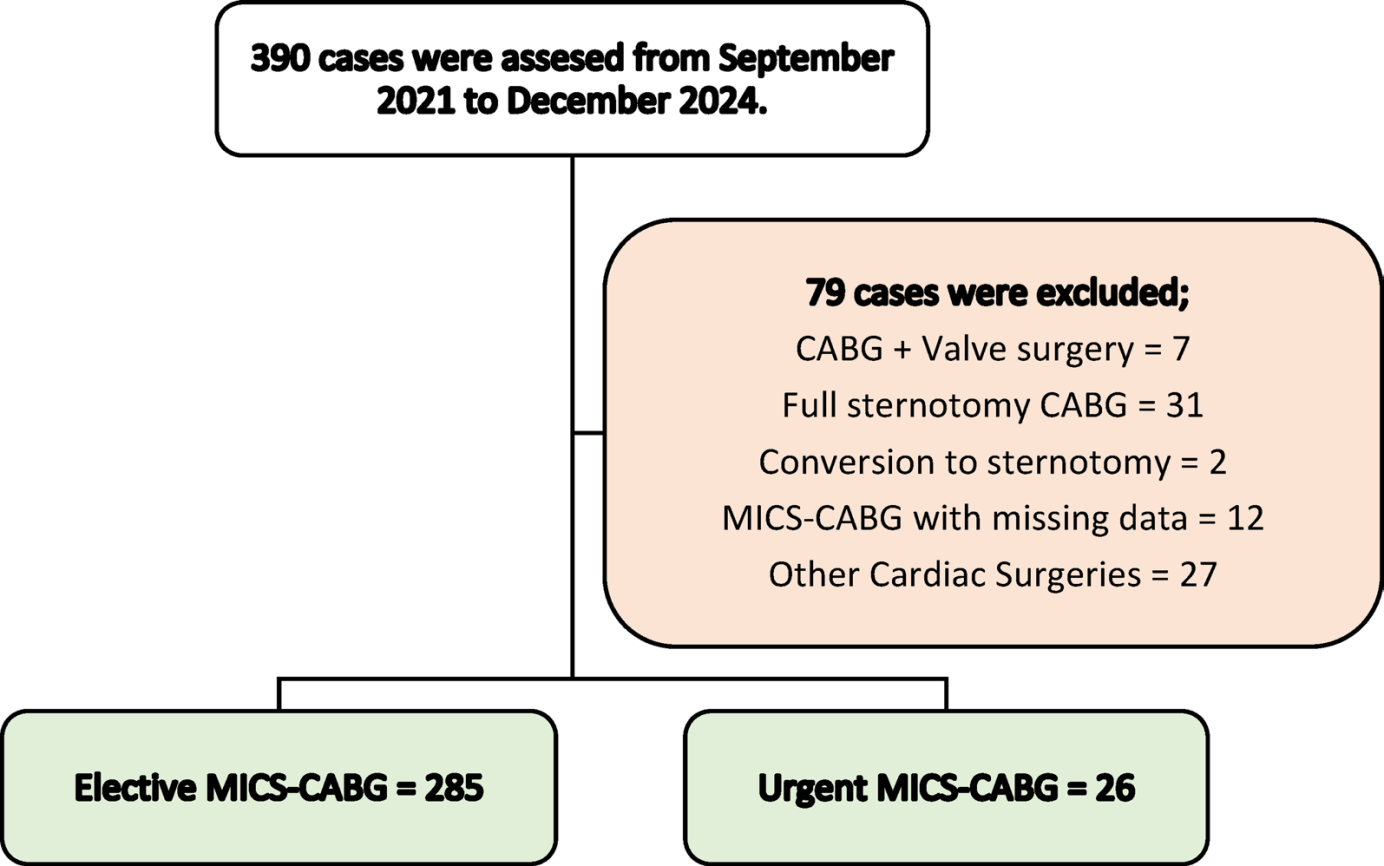
Study population flow diagram showing 390 cases assessed, 79 excluded, and 311 cases included: 285 elective and 26 urgent MICS-CABG cases

A total of 390 cases assessed during the study period, 311 cases met the eligibility criteria (elective group = 285 and urgent group = 26). Inclusion criteria were limited to patients who underwent isolated MICS-CABG. Patients were excluded if they underwent concomitant cardiac procedures (valve surgery, septal repair), full sternotomy, including emergent cases, to focus on isolated MICS-CABG or converted to sternotomy. Minimal missing data were excluded from analyses to preserve data integrity and ensure accurate study outcomes. The complete patient selection process, including the application of inclusion and exclusion criteria, is illustrated in Fig. 1.

Demographic characteristics, preoperative clinical profiles, angiographic findings, intraoperative details, and postoperative outcomes were extracted from the hospital’s medical records system and operative reports maintained by the cardiothoracic surgery unit.

## Variable and outcome definitions

Coronary stenosis was classified as significant if luminal narrowing was ≥ 70% in major epicardial vessels or ≥ 50% in the left main coronary artery, while critical stenosis was defined as ≥ 90% narrowing in any major vessel. For Post operative laboratory investigations, the most abnormal value, whether the highest or lowest as clinically relevant, was recorded for analysis. Postoperative outcomes were as follows: Acute respiratory failure (ARF) was diagnosed based on arterial blood gas findings, with hypoxemic ARF defined as PaO₂ < 60 mmHg on room air and hypercapnic ARF as PaCO₂ >50 mmHg with pH < 7.35. Myocardial infarction (MI) was defined by the presence of new ischemic ECG changes accompanied by a postoperative troponin rise ≥ 10 times the upper reference limit. Acute kidney injury (AKI) was classified according to KDIGO criteria as a serum creatinine increase of ≥ 0.3 mg/dL within 48 h. Postoperative bleeding was recorded as chest tube drainage exceeding 1,000 mL during ICU stay, regardless of whether reoperation was required.

## Surgical technique

All surgeries were performed by a single experienced cardiothoracic surgeon and his dedicated team, following standardized protocols for MICS-CABG. A 4 cm left peri-areolar incision was made at the 4th or 5th intercostal space, with an additional 10 mm port at the 3rd intercostal space in the left anterior mid-axillary line to facilitate endoscopic instrumentation. The left internal thoracic artery (LITA) was harvested endoscopically using a skeletonization technique with monopolar cautery, and the radial artery was harvested using a bridging technique with LigaSure. A LITA–radial artery loop graft was constructed, and the radial artery was sequentially anastomosed to the left anterior descending (LAD), obtuse marginal (OM), and posterior descending artery (PDA) as indicated. The majority of procedures were performed off-pump in hemodynamically stable patients, whereas cardiopulmonary bypass was utilized for selected on-pump cases. A left pleural drain was placed, and the chest was closed using standard techniques.

### Statistical analysis

Continuous variables were summarized as mean ± standard deviation (SD) with corresponding 95% confidence intervals (CIs), while categorical variables were reported as frequencies and percentages. Normality of continuous variables was assessed using the Kolmogorov–Smirnov test. Variables meeting normality assumptions were compared using the independent samples t-test, while those violating normality were analyzed using the non-parametric Mann–Whitney U test. Categorical variables were compared using the chi-square test. For contingency tables larger than 2 × 2 the Likelihood Ratio test was applied. When over 20% of expected cell counts in 2 × 2 tables were < 5, the Fisher’s exact test was used. All statistical tests were two-tailed, with a significance level set at *p* < 0.05. The specific test applied to each variable is indicated using superscript symbols in the corresponding tables. Statistical analysis was conducted using SPSS version 26.0 (IBM Corp., Armonk, NY, USA).

## Results

Patients were stratified into elective and urgent groups based on STS criteria. As shown in Table 1, no significant differences were observed in age, gender, or BMI between groups. Comorbidities such as triple-vessel disease, diabetes, and hypertension were similarly distributed.

**Table 1:** Baseline demographics and prevalence of chronic comorbidities in elective and urgent MICS-CABG patients

Variables	Elective group (*n* = 285)	Urgent group (*n* = 26)	*P* value
Age (years)	62.02 ± 8.74 (61.00–63.04)	59.08 ± 8.06 (55.82–62.33)	0.096
Gender (Male)	237 (76.2%)	19 (73.1%)	0.189
BMI (kg/m²)	29.56 ± 5.94 (28.87–30.26)	29.34 ± 4.75 (27.43–31.26)	0.747^¶^
Triple-vessel Disease	194 (68.1%)	19 (73.1%)	0.599
Diabetes Mellitus	194 (68.1%)	18 (69.2%)	0.903
Hypertension	129 (45.3%)	11 (42.3%)	0.772
Liver Disease	2 (0.7%)	0 (0.0%)	-
Chronic Lung Disease	5 (1.8%)	0 (0.0%)	-
Hypothyroidism	4 (1.4%)	1 (3.8%)	0.356^†^

All values are presented as frequency (percentage) or mean ± standard deviation with 95% confidence intervals (CI) in parentheses. BMI, body mass index. Statistical tests

¶ Mann–Whitney U test

† Fisher’s exact test

Table 2 presents a comparison of key preoperative laboratory and cardiac function parameters between patients undergoing elective and urgent minimally invasive coronary artery bypass grafting (MICS-CABG). Notably, the urgent group exhibited a significantly lower ejection fraction (49.2 ± 12.0% vs. 53.8 ± 10.0%; *p* = 0.032) and higher serum creatinine levels (1.59 ± 1.58 mg/dL vs. 1.19 ± 1.94 mg/dL; *p* = 0.008).

**Table 2:** Preoperative functional and laboratory parameters of elective and urgent MICS-CABG patients

Variables	Elective (*n* = 285)	Urgent (*n* = 26)	*P* value^¶^
Ejection Fraction (%)	53.8 ± 10.0 (52.4–55.0)	49.2 ± 12.0 (44.3–55.4)	0.032*
Serum Creatinine (mg/dL)	1.19 ± 1.94 (0.94–1.52)	1.59 ± 1.58 (0.94–2.45)	0.008*
Blood Urea (mg/dL)	38.6 ± 16.6 (36.2–40.3)	47.5 ± 23.7 (38.6–60.9)	0.078
Hemoglobin (g/dL)	13.58 ± 5.64 (12.89–14.56)	13.19 ± 2.05 (12.23–14.06)	0.634
White Blood cell (×10³/µL)	7.67 ± 1.94 (7.40–7.91)	8.21 ± 1.79 (7.35–8.92)	0.133
Platelet (×10³/µL)	229 ± 68 (219–238)	236 ± 73 (197–267)	0.241
HbA1c (%)	7.11 ± 1.53 (6.89–7.30)	6.98 ± 1.80 (6.33–7.93)	0.462
PT (second)	14.1 ± 0.8 (13.9–14.3)	13.3 ± 3.2 (13.7–14.5)	0.722
INR	1.15 ± 0.08 (1.14–1.17)	1.14 ± 0.10 (1.10–1.18)	0.714

All values are presented as mean ± standard deviation (SD) with 95% confidence intervals (CI) in parentheses. HbA1c, glycated hemoglobin; PT, prothrombin time; INR, international normalized ratio. Statistical tests

**¶** Mann–Whitney U test was applied for all comparisons

* Statistically significant at *p* < 0.05

The distribution pattern of coronary artery stenosis—including the posterior descending artery (PDA), right coronary artery (RCA), left main (LM), and left anterior descending artery (LAD)—was similar between the elective and urgent groups. No statistically significant differences were found in the severity (critical or significant) or presence of stenosis in any of the evaluated coronary vessels (*p* > 0.05). Full data are summarized in Table 3.

**Table 3:** Comparison of coronary artery stenosis severity between elective and urgent MICS-CABG patients

Coronary Artery Segment	Elective group (*n* = 285)	Urgent group (*n* = 26)	*P* value
**Posterior Descending Artery (PDA)**			0.966^‡^
- Critical Stenosis	84 (29.5%)	7 (26.9%)	
- Significant Stenosis	15 (5.3%)	1 (3.8%)	
- No Stenosis	182 (63.9%)	18 (69.2%)	
**Right Coronary Artery (RCA)**			0.989^‡^
- Critical Stenosis	162 (56.8%)	16 (61.5%)	
- Significant Stenosis	29 (10.2%)	3 (11.5%)	
- No Stenosis	44 (15.4%)	3 (11.5%)	
**Left Main (LM) Coronary Artery**			0.494^‡^
- Critical Stenosis	13 (4.6%)	1 (3.8%)	
- Significant Stenosis	4 (1.4%)	0 (0.0%)	
- No Stenosis	267 (93.7%)	25 (96.2%)	
**Left Anterior Descending (LAD)**			0.781^‡^
- Critical Stenosis	225 (78.9%)	20 (76.9%)	
- Significant Stenosis	21 (7.4%)	3 (11.5%)	
- No Stenosis	6 (2.1%)	0 (0.0%)	

All values are presented as frequency (percentage). Statistical tests

^‡^ Likelihood Ratio test

Table 4 summarizes intraoperative and postoperative parameters comparing elective and urgent MICS-CABG groups. While surgery duration and number of conduits used were comparable (*p* = 0.364 and *p* = 0.858, respectively), the urgent group had significantly higher cardiopulmonary bypass (CPB) usage (26.9% vs. 10.9%, *p* = 0.026). Use of vasopressor support was significantly higher in the urgent group, with increased administration of adrenaline (26.9% vs. 10.2%, *p* = 0.020) and noradrenaline (73.1% vs. 40.7%, *p* = 0.001). Postoperative serum creatinine levels were also significantly higher in the urgent group (1.86 ± 1.79 vs. 1.14 ± 0.58 mg/dL, *p* = 0.037), Additionally, white blood cell count was significantly higher in the urgent group (15.93 ± 3.70 vs. 14.31 ± 4.10 × 10³/µL, *p* = 0.045).

**Table 4:** Intraoperative and early postoperative parameters in elective and urgent MICS-CABG patients

Variable	Elective group (*n* = 285)	Urgent group (*n* = 26)	*P* value
Surgery Duration (hrs.)	4.67 ± 1.43 (4.49–4.85)	4.95 ± 1.42 (4.35–5.55)	0.364
CPB usage (On-pump)	31 (10.9%)	7 (26.9%)	0.026^†^*
CBP Time (mins.)	95.5 ± 45.9 (75.1–115.8)	104.7 ± 18.0 (85.7–123.6)	0.461^¶^
Number of Conduits	2.12 ± 0.49 (2.06–2.18)	2.21 ± 0.78 (1.88–2.54)	0.858^¶^
Adrenaline Use (%)	29 (10.2%)	7 (26.9%)	0.020^†^*
Noradrenaline Use (%)	116 (40.7%)	19 (73.1%)	0.001*
Ejection Fraction (%)	52.7 ± 9.3 (51.5–53.9)	51.9 ± 7.9 (47.7–56.1)	0.507^¶^
Serum Creatinine (mg/dL)	1.14 ± 0.58 (1.07–1.21)	1.86 ± 1.79 (1.10–2.61)	0.037^¶^*
Blood Urea (mg/dL)	48.1 ± 20.7 (45.6–50.6)	57.6 ± 28.7 (45.2–70)	0.259^¶^
White blood cell (×10³/µL)	14.31 ± 4.10 (13.81–14.82)	15.93 ± 3.70 (14.29–17.57)	0.045^¶^*
Hemoglobin (g/dL)	10.00 ± 1.66 (9.80–10.20)	9.43 ± 1.79 (8.65–10.20)	0.111^¶^
Platelets (×10³/µL)	184 ± 61 (177–192)	181 ± 51 (159–204)	0.743^¶^

All values are presented as frequency (percentage) or mean ± standard deviation with 95% confidence intervals (CI) in parentheses. BMI, body mass index. Statistical tests

¶ Mann–Whitney U test

† Fisher’s exact test, * Statistically significant at p < 0.05

Table 5 compares the incidence of postoperative complications between elective and urgent MICS-CABG groups. Although ICU mortality appeared higher in the urgent group (11.5% vs. 2.8%), the difference did not reach statistical significance (*p* = 0.056). Acute respiratory failure was significantly more frequent in the urgent group (39.1% vs. 17.5%, *p* = 0.023). Other complications, including AKI, myocardial infarction, ICU readmission, and hospital stay duration, were not significantly different between the groups.

**Table 5:** Postoperative complications in elective vs. Urgent MICS-CABG patients

Complications	Elective group (*n* = 285)	Urgent group (*n* = 26)	*P*-value
ICU Mortality	8 (2.8%)	3 (11.5%)	0.056^†^
ICU Stay (hours)	16.9 ± 12.6 (15.4–18.4)	17.4 ± 8.2 (14.1–20.7)	0.544^¶^
Readmission to ICU	2 (0.7%)	1 (4.3%)	0.215^†^
Hospitalization (Days)	5.30 ± 2.09 (5.06–5.55)	6.14 ± 3.05 (4.86–7.43)	0.167^¶^
Blood Loss > 1000 mL Without Reoperation	18 (6.6%)	2 (8.7%)	0.660^†^
Myocardial Infarction	7 (2.6%)	0 (0.0%)	-
Acute Respiratory Failure	48 (17.5%)	9 (39.1%)	0.023^†^*
Acute Kidney Injury	32 (11.7%)	5 (21.7%)	0.183^†^

All values are presented as frequency (percentage) or mean ± standard deviation (SD) with 95% confidence intervals (CI) in parentheses

¶ Mann–Whitney U test

† Fisher’s exact test, * Statistically significant at p < 0.05

## Discussion

In our study, we observed clear differences in perioperative and postoperative outcomes between urgent and elective MICS-CABG patients, with surgical urgency emerging as a significant determinant of adverse events. The most prominent differences included a higher reliance on intraoperative vasopressor support and a greater incidence of acute respiratory failure in urgent cases. These findings underscore the physiological burden that urgency imposes, particularly in the context of compromised hemodynamics and reduced cardiorespiratory reserve. Previous studies have consistently shown that urgent cases carry increased risks due to the limited time available for preoperative optimization and the urgent nature of the patient’s condition [11, 12].

The preoperative ejection fraction was significantly lower in the urgent group, suggesting that ventricular dysfunction may contribute both to the need for urgent intervention and to poorer postoperative trajectories. A reduced EF has been linked to impaired organ perfusion and reduced physiological reserve, which heightens susceptibility to complications such as renal injury and pulmonary dysfunction [13, 14]. In urgent settings, the inability to optimize EF preoperatively further compounds these risks, potentially influencing both surgical decision-making and intraoperative management.

Longer operative times in urgent cases, despite a similar number of conduits used, likely reflect greater procedural complexity and intraoperative instability. The markedly higher use of adrenaline and noradrenaline in the urgent group aligns with previous reports, such as Mohammad et al., who found that urgent CABG for acute coronary syndromes often demands aggressive inotropic and vasopressor support to maintain perfusion pressures [11].

Urgent patients showed higher postoperative serum creatinine and BUN, reflecting not only perioperative renal stress but also possible preoperative compromise. In urgent MICS-CABG, renal perfusion may already be reduced by acute coronary syndrome, cardiogenic shock, or instability, leaving minimal reserve before surgery. Intraoperative factors—cardiopulmonary bypass, inflammation, and vasopressor-induced vasoconstriction—can then trigger a “two-hit” injury. Even without statistically significant AKI rates, these biochemical changes are relevant, as transient creatinine rises predict worse long-term outcomes. Consistent with our findings, Scherner et al. reported that urgent CABG significantly increases AKI incidence and severity, with preoperative renal impairment a key predictor [15].

Acute respiratory failure was significantly more common in the urgent CABG group, indicating a higher burden of postoperative pulmonary complications in this subset. Urgent revascularization is frequently performed in patients with acute coronary syndrome or decompensated heart failure, conditions often accompanied by elevated pulmonary capillary wedge pressure, pulmonary congestion, and reduced respiratory reserve. Hemodynamic instability in these patients may necessitate the use of high-dose vasopressors, which are known to cause pulmonary vasoconstriction and increase capillary permeability, thereby exacerbating ventilation–perfusion mismatch and oxygenation impairment. Sarkar et al. highlights that catecholamine vasopressors can increase pulmonary vascular resistance and impair oxygen exchange [16], and Vickneson et al. identified hemodynamic instability and urgent operations as independent predictors of major complications, including pulmonary dysfunction, in low EF patients undergoing CABG [17].

The findings of this study have important implications for the management of patients undergoing MICS-CABG, particularly in urgent settings. As this is a single-center study conducted in Iraq, the results should be interpreted in the context of the local healthcare infrastructure, perioperative protocols, and ICU management practices, which may differ from those in higher-resource settings. Variability in surgical expertise, postoperative monitoring capacity, and access to advanced perioperative optimization strategies could influence outcomes and limit generalizability.

Future research should address baseline differences—particularly the lower preoperative ejection fraction, higher creatinine levels and higher frequency usage of cardiopulmonary bypass in urgent cases—using methods such as propensity score adjustment or matching to reduce selection bias and improve validity. Prospective designs or well-matched retrospective studies are needed to better isolate the effect of surgical urgency. Targeted areas include rapid preoperative risk stratification, and optimized perioperative hemodynamic support.

## Strength and limitations

While outcomes of urgent CABG have been explored in conventional sternotomy cohorts, evidence in the setting of MICS-CABG remains scarce. This study addresses this gap by providing one of the few direct comparisons between urgent and elective MICS-CABG cases, thereby contributing novel insights into perioperative management and early outcomes in this surgical context. This study has limitations. Its retrospective design introduces potential selection bias and incomplete data capture. The urgent group comprised only 26 patients (8.4%), limiting statistical power, especially for secondary outcomes like ICU mortality, AKI, and readmission. As a single-center study, results may not generalize to settings with different surgical expertise, perioperative protocols, or resources. Additionally, long-term outcomes such as survival, graft patency, and quality of life were not assessed and should be explored in future investigations.

## Conclusion

Urgent MICS-CABG was associated with higher risks of renal dysfunction, respiratory failure, and increased vasopressor use than elective cases, largely due to preoperative instability and limited optimization.

## Data Availability

No datasets were generated or analysed during the current study.

## Abbreviations

CABG: Coronary artery bypass graft
MICS-CABG: Minimally Invasive Cardiac Surgery Coronary Artery Bypass Graft
ICU: Intensive care unit
AKI: Acute kidney injury
